# Mining Boom, Labour Market Segmentation and Social Inequality in the Congolese Copperbelt

**DOI:** 10.1111/dech.12531

**Published:** 2019-07-03

**Authors:** Benjamin Rubbers

## Abstract

The study of the impacts of new mining projects in Africa is generally set in a normative debate about their possible contribution to development, which leads to a representation of African societies as divided between beneficiaries and victims of foreign investments. Based on research in the Congolese copperbelt, this article aims to examine in more detail the inequalities generated by the recent mining boom by taking the processes of labour market segmentation as a starting point. It shows that the labour market in the mining sector has progressively been organized along three intersecting lines that divide it: the first is between employment in industrial and artisanal mining companies, the second is between jobs for mining or subcontracting companies and the third is between jobs for expatriates, Congolese skilled workers and local unskilled workers. Far from simply reflecting existing social inequalities, the labour market has been actively involved in their creation, and its control has caused growing tensions in the Congolese copperbelt region. Although largely neglected in the literature on extractive industries, processes of labour market segmentation are key to making sense of the impacts of mining investments on the shape of societies in the global South.

## INTRODUCTION

During the last two decades, African countries endowed with mineral resources have witnessed an unprecedented rise in foreign direct investments in the mining sector, primarily from Europe and North America but also, for a rapidly growing share, from China. It is well‐documented that this boom was largely the result of the liberalization of the African mining sector that has been promoted by the World Bank as a new development strategy since the 1990s. Following this liberalization process, hundreds of companies flocked to resource‐rich countries to obtain mining rights and to start new projects.

The implementation of these neoliberal reforms has spawned a large amount of literature which has challenged the World Bank's assumptions about the contribution of mining to development (for an overview, see Bebbington et al., 2008; Pegg, 2006). On the basis of aggregate figures, proponents of the resource‐curse thesis have argued that the abundance of natural resources in poor countries does not generally bring expected benefits; on the contrary, it is correlated with poor development indicators and the occurrence of armed conflicts (Collier and Hoeffler, 2005; Ross, 1999; Rosser, 2005; Sachs and Warner, 1995). At the local level, a multitude of reports by non‐governmental organizations (NGOs) have documented the weak and/or negative impacts of industrial mining projects in terms of poverty alleviation. In the academic literature on Africa, the critique has, to a large extent, focused on the marginalization of artisanal miners, and on how mining companies deal with them (see, e.g., Carstens and Hilson, 2009; Geenen and Verweijen, 2017; Hilson, 2002, 2009; Hilson and Haselip, 2005; Hilson and Yakovleva, 2007).

The conclusion of most of these studies is that mining reforms have only benefited the few, leaving the local population behind: the rise of foreign investments in the industrial mining sector has led to growing inequalities between a minority of ‘winners’ (for example company shareholders, political authorities, local elites, etc.), and a majority of ‘losers’ (including artisanal miners and local communities and individuals). In this respect, the literature echoes those scholars who see behind neoliberal reforms a project of ‘accumulation by dispossession’ (Harvey, 2003: 137–82) which generates a growing ‘relative surplus population’ — a population deprived of its means of subsistence and, at the same time, of no use to capital (Li, 2009, 2011, 2017; Sassen, 2010). While this line of analysis is probably correct in general (a minority receives a disproportionate share of the benefits), it remains far too simple. This article aims to study the inequalities generated by the mining boom in more detail by taking the ‘segmentation’ of the labour market in the copper mining sector in the Democratic Republic of Congo (DRC) as a point of departure.^1^

The study of labour market segmentation was originally developed by Doeringer and Piore (1971) to account for the persistence of racial and social inequalities among American workers in the 1960s. For these authors, such inequalities were the result not only of people's career strategies, but also of the organization of the labour market. Far from constituting a homogeneous space responding to the law of supply and demand, the labour market is viewed as being divided into two segments with different recruitment procedures, different wages, different working conditions, and different career opportunities: a primary segment, corresponding to the ‘internal market’ of large firms, and a secondary segment, that is associated with the low‐paid, precarious jobs that are offered by small firms. As poor (black) workers are largely excluded from ‘good’ jobs in the primary segment, this dual organization of the labour market contributes to the creation of racial and social inequalities. Since this seminal work, labour segmentation theory has been developed in political economy, sociology and geography, to better take into account:

1) The multiple ways in which the labour market can be segmented: these are no longer represented as founded on a simple primary/secondary opposition depending on the size of firms (Jones, 1983; Michon, 1987);
2) The various factors involved in segmentation independently of technical and economic constraints: employers’ control strategies, state regulations, the role of trade unions and other pressure groups, and so on (Gordon et al., 1982; Kalleberg and Sorensen, 1979; Kenny and Webster, 1998);
3) The spatial dimension of labour markets, and their embeddedness in various forms of social life (Peck, 1996).

This body of literature provides a possible approach to account empirically for the persistent salience of race, gender, class and other forms of division (religion, ethnicity, etc.) in the organization of global capitalism's relations of production (Brodkin, 2000; see also Tsing, 2009).^2^

After a brief description of the organization of the labour market before the liberalization of the mining sector in the early 2000s, I will attempt to outline the ‘organizational segmentation’ — that is, the working conditions found in different types of companies — of the labour market that followed the establishment of foreign mining investors in the copperbelt region. I will then discuss in more detail the recruitment practices of mining companies according to the type of job — what might be called ‘the occupational segmentation’ of the labour market. Finally, I will detail a case study on one specific company to bring to light the tensions resulting from inequalities in the labour market and their spatial embeddedness. Generally speaking, the article will focus on the forms of segmentation of the labour market, and their outcome in terms of economic and social inequalities. For reasons of space, the various factors involved in these processes (the mode of production, control strategies, state regulations, the role of unions, etc.) will be left in the background. They will be the subject of another article on the micro‐politics of work in the mining sector.

The Congolese copperbelt provides a particularly interesting case through which to study inequalities in the labour market in the context of the mining boom. For more than a century, living in the urban centres of this region has been closely associated with the opportunity to work and, even today, access to employment is one of the most pressing claims that people make from mining companies. Moreover, over the last 20 years, the copperbelt has attracted investors of various sizes and origins who have contributed to the development of both industrial and artisanal mining. Understanding the changes that they have made in the domain of labour implies, therefore, moving beyond the tendency in the literature on extractive industries to focus on one project, or on investors from one country, in order to study the dynamics of the labour market more broadly.

The Congolese mining sector nevertheless poses serious challenges to researchers. The only available figures on employment in the mining sector are those provided by the Extractive Industries Transparency Initiative (EITI): Appendix 6 of the organization's 2014 and 2015 reports give an indication of the number of direct and indirect jobs created by mining companies for expatriate and national workers.^3^ It is almost impossible for researchers to complement or update these figures given the large number of companies involved, the difficulty of accessing those established in rural areas and the reluctance of employers to disclose information. The analysis below is principally based on research carried out between 2016 and 2018 within the framework of the WORKinMINING project — a comparative research project on labour in Zambia and DRC funded by the European Research Council. Over the course of several fieldtrips, I undertook approximately 100 interviews with the human resources (HR) managers of more than 20 mining companies, as well as trade unionists, workers, subcontractors, political figures, labour inspectors and customary chiefs — all those involved in the regulation of the labour market broadly speaking. The resulting data are very uneven: they are detailed for some companies, but much less so for others. However, complemented by past research, advocacy organizations’ reports, and data collected by other project team members in the DRC, the fieldwork provides — I believe — a comprehensive view of the dynamics of the labour market in the mining sector as a whole.

## AN INTERNAL LABOUR MARKET

For many years the mining sector in the Congolese copperbelt region was dominated by the state‐owned companies Union Minière du Haut‐Katanga (UMHK), from 1906 to 1967, and Générale des Carrières et des Mines (Gécamines), from 1967 to 2003. In the early colonial period, UMHK depended on contractors who recruited migrant workers from afar. In the 1920s, in order to overcome the shortage of labour, UMHK implemented a ‘stabilization’ programme that consisted of organizing its own recruitment missions in rural areas and modernizing its camps to allow workers to come and live there with their wives and children (Dibwe dia Mwembu, 2001; Fetter, 1973; Higginson, 1989; Perrings, 1979). Workers recruited through these missions — with a significant contingent coming from the Kasaï province — came to form the core of its workforce: the company took charge of every aspect of their lives, and established an internal labour market that prioritized them, their male children and their parents. This policy was continued after the DRC gained independence in 1960. Although Gécamines put in place a more open recruitment procedure (Henk, 1988), a large number of job candidates came from the company's schools and families, so it did not deter the social reproduction of the workforce.

Following the collapse of Gécamines's Kamoto underground mine in 1990, the company's production decreased abruptly. This decline in production was not immediately followed by staff cuts, although the political mobilization to expel the ‘Kasaïens’ from Katanga province in 1992**–**93 resulted in the departure of approximatively 9,000 workers (from a total of 34,000) from the company (see Dibwe dia Mwembu, 2001). On the other hand, confronted by hyperinflation, wage arrears and disruptions in the distribution of food rations, Gécamines's workers experienced a rapid deterioration in their living conditions. Plunged into extreme poverty, they had no choice but to develop informal activities in subsistence agriculture, petty trade, or poultry farming with their wives and children (Rubbers, 2013).

To address this situation, the World Bank pushed the state‐owned company to transfer some of its mining and industrial assets to joint‐venture projects with foreign investors. Although the first partnership contracts were signed in the second half of the 1990s, most were concluded after the adoption of the new mining code in 2002, which provided particularly attractive conditions for investors. In some cases, Gécamines employees were also transferred to joint‐venture projects through an employer substitution procedure. To allow Gécamines to continue its own operations on a smaller scale, the World Bank put in place a restructuring plan that involved reducing the company's personnel from 24,000 to 14,000 workers. This reduction in staff was organized within the framework of a controversial ‘voluntary departure programme’ in 2003**–**04 (Rubbers, 2010, 2017).

The recovery plan has not met its objectives so far. In 2015, independently of the joint‐venture projects in which it is involved, Gécamines produced only 18,000 tons of copper — 2 per cent of the total production (1 million tons) in DRC and 4 per cent of its record production level (486,000 tons in 1986). However, with 8,600 workers, it remained the largest employer in the copperbelt's mining sector, which employed a total of 40,000. Compared to those in new mining projects, these workers have enjoyed greater job security (there were no redundancies at Gécamines following the fall in copper prices in 2008**–**09 and 2011**–**15) and a larger range of benefits, including free access to schools for their children. On the other hand, their wages are much lower than those paid in the large private mining companies and they had, until recently, suffered several months of wage arrears. This is why those who are offered the opportunity do not hesitate to leave Gécamines for the new joint‐venture projects.

## ORGANIZATIONAL SEGMENTATION

Since the late 1990s, the mining sector in the Congolese copperbelt has undergone a dual process of liberalization: from above, with the creation of new industrial mining projects, and from below, with the development of artisanal mining. On the one hand, as mentioned in the previous section, Gécamines was forced by the World Bank to make several of its mines available for foreign investors. Under the mining code, these mines had to be sold, but due to political interference, the majority of new mining projects have been set up as joint ventures with the state‐owned company as a minority partner (Carter Center, 2017). The latter is currently bound by 30 partnership contracts, and only two leasing contracts. From this total of 32 projects, 14 are currently in the production phase. These involve various types of investors: Western majors and juniors, Chinese state‐owned companies, and smaller private firms of various origins. On the other hand, in 1998, Laurent‐Désiré Kabila's government asked Gécamines to make some quarries available for artisanal mining. Within a few months, tens of thousands of artisanal miners had entered a number of the company's quarries, a marketing chain for heterogenite (cobalt‐rich ore) was put in place, and the plants (both smelters and hydrometallurgical plants) to process this ore were built (see Rubbers, 2013). The first plants were created by Greek and Indian businessmen who were already established in DRC. In the mid‐2000s, they were followed by Chinese migrant entrepreneurs, who started building up to 30 small processing units in various parts of the copperbelt.^4^ Following the financial crisis in 2008, most of these enterprises closed down (Cuvelier, 2009; Jansson, 2012). Since then, fewer than a dozen enterprises have resumed their operations. While the smallest among them continue to exclusively buy heterogenite from dealers, traders and artisanal miners, the largest — medium‐sized enterprises that are owned by Indian and Chinese businessmen — have also managed to obtain rights over mines that they develop industrially (with machines) or semi‐industrially (with machines and artisanal miners).

While artisanal mining provides a means of subsistence for a large number of people in the copperbelt region, the reform of the mining sector designed by the World Bank has done nothing to support this sector. Since the early 2000s, most of the mines in which these people were working have been transferred to foreign companies, which have taken strong measures to expel the miners from their new property. These forced evictions have led to a number of conflicts over the past 15 years, as a large number of artisanal miners have continued to enter the mining concessions illegally. While the mining code provides for the creation of ‘artisanal mining zones’, the government has been reluctant to delineate them on the map. It was only in 2014 that 78 of these zones were created in the copperbelt. By the Director of the mining cadastre administration's own admission,^5^ most are actually inadequate for artisanal mining. Unless they encroach illegally on industrial mining concessions, artisanal miners are thus increasingly left with a choice between moving to small mining sites far from urban centres, or working for semi‐industrial companies as day labourers.

In view of this dual process of liberalization, the labour market in the mining sector can be divided into two broad segments corresponding to the distinction between industrial and artisanal companies. Industrial mining companies offer permanent jobs, high wages and good working conditions. Progressively, they have established a bureaucratic organization of work, including industrial relations, which comply with Congolese law. In artisanal mining companies, by contrast, most workers are recruited as unskilled day labourers and earn very low wages. Their organization of work, which is more dependent on personal relationships with management, is to varying degrees characterized by unfair dismissals, poor health and safety conditions, and lack of union representation. Such violations of the law are hidden by management who buy the silence of labour inspectors.

Following Doeringer and Piore's thesis (1971), the distinctive labour policies of industrial and artisanal mining companies are best understood in the light of their differences in size, the importance of their capital, their technological development, their level of production, their number of employees, etc. From this perspective, the widely held opinion that Asian companies offer inferior conditions of employment compared with those offered by Western companies is correct only insofar as most of them are small mining companies processing ore from artisanal mining. Several human rights organizations (such as Action Against Impunity for Human Rights, Premi Congo, etc.) have published reports denouncing the working conditions in these small enterprises. It is, however, important to add that conditions of employment in the small companies created by Greek businessmen in the early 2000s were not much better.

For industrial mining companies, the situation is more complex, as several mining projects have been developed by Western companies and then bought out by Chinese enterprises. Since these enterprises have kept the existing management structures in place, they offer analogous conditions of employment to those of Western mining projects. In this article, therefore, following local usage, these companies with capital from China and managers from North America, South Africa or Australia are included in the category of Western companies. It is true that companies with capital and management from China — such as the Sino‐Congolaise des Mines project (SCM)^6^ — mostly hire unskilled workers, and offer them lower wages than the Western mining companies. However, these projects only began production in the 2010s, when copper prices were low. Unlike Western investors, SCM was not able to take advantage of the commodity super‐cycle. Whether in artisanal or industrial mining projects, poorer conditions of employment in Chinese companies can be explained by factors other than the nationality or culture of capital (Hairong and Sautman, 2013; Mohan, 2013). In addition, the contrast between Asian and Western companies underestimates the capacity of smaller Asian enterprises to improve their employment practices (Lee, 2017). The history of the company Chemicals of Africa (CHEMAF), owned by an Indian businessman, is a case in point:

This enterprise started in 2002 with a hydrometallurgical plant that processed ore bought from artisanal miners, before it obtained rights over several mines. For a long time, the bulk of the workforce was composed of day labourers who worked under the control of Indian and Congolese supervisors. The management of the company was marked by the arbitrary power of its owner, and the struggles of the small informal networks that evolved in his entourage. Confronted with threats of strike action and the payment of high penalties to the labour inspectorate, however, efforts have been made in recent years to improve working conditions and formalize the management of human resources: the number of permanent employees has been increased, and their salaries augmented; the employment of day labourers has been outsourced to a subcontractor; and various rules and procedures have been adopted. In 2015, CHEMAF reported employing 1,104 direct workers, including 152 expatriates, and 689 indirect workers to the ITIE. According to the HR manager, Congolese (direct) workers earn, on average, one third less than those working for Western mining companies, but receive equivalent benefits.^7^

The growing use of outsourcing among mining companies has introduced another form of duality in the labour market that cross‐cuts the distinction between the labour markets created by artisanal and industrial mining companies. Independently of the market for direct jobs (in mining companies), a large market for indirect jobs (in subcontracting companies) has developed since the beginning of the mining boom. Outsourcing is general practice for all mining projects in the 21st century, from exploration to production. Since it is one of the principal methods by which mining companies cut costs, however, the downturn in copper prices in 2008, and again in 2011, gave it a new impetus. In several projects, those tasks that were previously performed by employees (cleaning, catering, training, etc.) have been outsourced over the past decade. In contrast to the situation in Zambia (Hairong and Sautman, 2013; Lee, 2017), Chinese enterprises are no exception to this trend in DRC.

As subcontracting includes various types of actors (multinational companies, middle‐sized foreign firms, Congolese businessmen, etc.) in different sectors, it is impossible to provide a general picture of the market for indirect jobs. However, three broad differences to the direct labour market can be identified: (1) it involves a much larger proportion of unskilled temporary jobs (the type of work that mining companies outsource in the first place); (2) whatever the level of skills, jobs on this market are more precarious (they depend on contracts with mining companies); and (3) salaries are generally inferior, although this is not always the case; in multinational companies and some middle‐sized enterprises, they are comparable to those paid by large mining companies. In the same way, casual workers in small Chinese enterprises receive more or less the same wage as the permanent unskilled workers (between US$ 100 and US$ 200; see below).

Although artisanal mining is to a large extent a subsistence activity, it is certainly not disconnected from the circuits of capitalism. On the contrary, it is an integral part of the global cobalt supply chain. Accordingly, artisanal miners, traders and dealers can also be seen as subcontractors of the mining industry. Artisanal mining is the principal source of supply of small plants processing heterogenite. It is also the mode of production that some small and medium‐sized enterprises have chosen in order to develop their mines: they work with a cooperative of artisanal miners, or hire them as day labourers through a labour broker. Finally, some industrial mining companies buy copper and cobalt oxides from traders to upgrade and/or complement their plant's supply. Generally speaking, artisanal miners can be viewed as part of a large labour submarket — a market subordinated to the global mining industry, but organized independently, and characterized by poor working conditions (long working hours, poor health and safety conditions, irregular earnings, etc.). In total, this submarket produces more than 20 per cent of DRC's cobalt.

In 2016, Amnesty International and African Resources Watch started a media campaign that attracted public attention to this subcontracting relationship. Their report warned of human rights abuses, including child labour, that were linked to cobalt production in the artisanal mines of the copperbelt (Amnesty International, 2016). In response, the Chinese Chamber of Commerce for Metals, Minerals and Chemicals Importers and Exporters, which counts major car manufacturers and electronics companies among its members, immediately launched the Responsible Cobalt Initiative, which aims at establishing a cobalt supply‐chain management system in accordance with the Organization for Economic Cooperation and Development due diligence guidelines. A cobalt traceability pilot project using blockchain technology is under way at the time of writing this article. It is too early to assess the results of this initiative, but if it is implemented effectively, it is likely to have the same negative effects as the various initiatives established to control the 3Ts (tin, tungsten and tantalum) supply chains in Eastern Congo: it will contribute to the further marginalization of artisanal miners for the benefit of mining companies and the state (see Diemel, 2018; Diemel and Cuvelier, 2015; Seay, 2012; Vogel and Raeymaekers, 2016).

According to the most recent EITI report, in 2015 there were 50 mining companies in the copperbelt region.^8^ Together, they (including Gécamines) created 40,000 direct jobs and at least 22,000 indirect jobs.^9^ There are no reliable figures for the number of artisanal miners, which varies according to seasons and cobalt prices. One of the most serious studies on this issue (Tsurukawa et al., 2011) suggests that between 67,000 and 79,000 people were employed in artisanal mining in 2011 (74 per cent as miners and others as washers, porters, etc.). Although this number has probably declined since then, artisanal mining is still firmly established in the copperbelt (see Faber et al., 2017).

## OCCUPATIONAL SEGMENTATION

The representation of the labour market as divided into two broad segments according to the size or type of firms (organizational segmentation) fails to take into account variations in job quality within firms (occupational segmentation). In the case of mining investments in the global South, one must pay particular attention to the differences in recruitment practices for expatriate executives and technicians, national skilled workers and local unskilled workers. These recruitment channels correspond to significant disparities in terms of hierarchical position, job security and the level of remuneration. This three‐level structure is transversal to all companies, even though the number of workers in each segment varies according to the company's size and origins.

### Expatriates

Throughout the 20th century, the labour market for white workers remained separate from that for black workers. In the early days, most expatriates were recruited in the United Kingdom, or from among migrants from Southern Africa. After control of UMHK passed into Belgian hands in the late 1910s, however, Anglophone workers were progressively replaced with young graduates from the metropole (Fetter, 1973; UMHK, 1956: Ch. 2). The number of Belgian employees grew with the development of UMHK's operations after World War II. It was only after the company was nationalized, and a programme to Africanize management personnel was put in place, that this number gradually dropped from about 2,000 in the 1960s to 500 in the late 1980s. The liberalization of the mining sector and the boom of foreign investments in the early 2000s, however, contributed to a revival in the market for expatriate workers in DRC.

Expatriates are usually recruited as executives or technicians by the joint‐venture majority partner's main or regional office in the country in which it is established. In global mining companies, a certain number can also be transferred from other projects around the world, or recruited from the international market through specialized sites or agencies. In DRC, this is a market to which the mining project's HR manager (a position reserved for nationals) does not have access, as one manager explained:

The law provides that the company can only hire an expatriate if no Congolese candidate for the position can be found, but the way we recruit here, expatriates fall like rain [i.e. in a sudden and unpredictable way]. I often find new expatriates on site: ‘Hello, since when?’. They are in positions that could have been occupied by Congolese people, but I can't do anything. I have not even been informed of the opening of the position.^10^

For a certain number of jobs, the choice to recruit an expatriate can be justified by that person's specific skills. Generally, however, the main office abroad does not even inquire about the existence of workers with the required skills on the Congolese market: they directly hire someone from abroad. In addition, skills cannot account for the recruitment of all expatriates: I met expatriates whose work as supervisors could have been performed by nationals; some Congolese workers even told me that they had to train the expatriate who later became their superior. The choice to hire an expatriate instead of a Congolese is as much based on social and cultural as on professional criteria: expatriate executives prefer to hire people with a recognized degree, who speak their language, whom they feel they can trust and, if possible, with whom they have already worked. These are the main reasons given to justify the recruitment of expatriates, even though they cost the company more than nationals.

In Indian and Chinese companies, the cost of employing expatriates is not a problem, as the monthly salary of Asian expatriates — which ranges from US$ 1,000 to US$ 3,000 — is generally not much higher than that of Congolese managers with similar qualifications. This is much lower than the salaries of expatriates recruited on the international market, who earn from US$ 6,000 to US$ 25,000 per month; in addition, they are offered regular home leave, while Indian and Chinese workers must often wait for the end of their contract to take holidays.

In most companies, expatriates sign two‐year contracts, the duration of which corresponds to the validity of their work permits in DRC. At the end of this period, they are never sure that their contract will be renewed, and it is not necessarily easy to find such a well‐paying job elsewhere. In addition, expatriates — who often sign contracts in their home country — are easily dismissed. When copper prices declined in 2008**–**09, and then again in 2011**–**16, cuts in the expatriate staff were made in most companies before, or at the same time as, cuts in the number of national staff.

Few Congolese workers manage to access the positions occupied by expatriates.^11^ To do so, ideally they must be fluent in English, have obtained a university degree abroad, and/or introduce themselves through expatriate personal networks — which is difficult to do when expatriates tend to stick together. An increasing number of Congolese workers with several years of work experience in the sector, however, are being recruited by head hunters established abroad, or through a specialized website, to work for mining projects in DRC. They are offered salaries that are higher than those of their compatriots, but significantly lower than those of the majority of expatriates — that is, between US$ 4,000 and US$ 10,000 per month.

Legally, the number of foreign workers cannot exceed 10 per cent of the total workforce. However, this percentage is exceeded in many Asian companies, which tend to put Indian or Chinese workers in all positions involving responsibility. While SCM, for example, has signed a special agreement with the Congolese state that allows it to do so, smaller artisanal mining companies generally bribe low‐level officials so that they turn a blind eye to the number of expatriates. Although Western companies generally comply with the law in this domain, the total number of expatriate workers on site often exceeds 10 per cent if those working for service or consultancy companies are taken into account. In some projects, the foreign staff is more important in subcontracting than in the permanent workforce.

### Congolese Skilled Workers

The recruitment process involving Congolese managers and skilled workers differs between Western and Asian companies. In Western companies, it is undertaken by the HR departments of the mining projects (not the foreign joint‐venture partner's office), which publicize job vacancies through the National Employment Office and media outlets such as Okapi radio, MediaCongo's website, FaceBook, etc. In Asian companies, news of job vacancies circulates almost exclusively amongst workers via word of mouth. In both types of company, however, most candidates for skilled jobs find the information through personal networks — principally former colleagues and friends from university — and come from the copperbelt's largest cities (Kahola, 2015). The labour market in the Congolese mining sector is not very different from other labour markets in this respect (Granovetter, 1995).

In contrast to Chinese companies, Western companies have put in place a formal selection procedure: candidates are first selected by the HR department on the basis of their curriculum vitae (CV); those who have obtained the best test results are then interviewed by a committee of HR and recruitment professionals, who make the final choice. Amongst the skilled workers I met, few were offered their job without at least passing an interview. Yet this examination procedure causes widespread suspicions of influence peddling, patronage and tribalism. After all, managers involved in the process have plenty of room for manoeuvre to favour or exclude candidates: the HR manager, who receives recommendations from all sides (family members, customary chiefs, political authorities, etc.), has the opportunity to sort CVs; at the end of the process, it is traditionally the head of the department in which the candidate will work who has the final say. Finally, both managers can see their influence nullified by instructions from the executives above them.

In all mining companies, Congolese skilled workers are offered permanent contracts. However, this does not mean much in a context where companies see labour as a cost variable to be adjusted to changes in copper prices. According to Cuvelier (2009: 10–11), following the financial crisis of 2008, several Chinese enterprises processing ore from artisanal mining left the DRC, and their workers lost their jobs overnight. In the same period, and then again between 2011 and 2015, most Western mining companies made staff cuts through various procedures such as mass redundancy, voluntary departure programmes or technical leave. However, this is not the case with all mining projects. Despite the challenges confronting them, there are mining companies of various sizes and origins that have not reduced their workforce numbers. This is a decision that depends less on the type of investor than on the constraints within which they are working: the project's development phase, production costs, the joint‐venture majority partner's liabilities, etc. For now, the evolution of the labour market in DRC does not really allow us to corroborate Lee's thesis (2017) of a contrast between the short‐term perspective of private global capital and the more long‐term perspective of Chinese state capital.

At the same time, and especially during the boom years (2004–11), mobility of the skilled workers themselves between companies has increased. As interviews show, they no longer hesitate to resign in order to seek a better job (with a higher salary and/or closer to their family) elsewhere. It must be said that, unlike Gécamines in the past, mining companies offer their workers few opportunities for career advancement, even if they provide them with access to training programmes abroad. So, to climb the ladder, workers must apply for a job in another company. Since the downturn of copper prices in 2011, however, inter‐firm mobility has slowed considerably (the personnel turnover ratio is very low in all the companies for which I could obtain figures). The workers I met told me that they tried to keep their jobs while waiting for the mining sector to recover.

Linked to each other by various types of social network, the workers of different mining companies constantly compare their conditions of employment. Although small in number and length, the strikes that have broken out in the last decade have generally been caused by differences in salaries, bonuses and benefits between companies. On a more regular basis, union representatives readily use this type of comparison, and the risks of strikes associated with them, to demand improvements from employers. Since the beginning of the mining boom, this risk of strikes, coupled with inter‐firm competition for skilled and experienced workers, has led to a gradual upward convergence of wages across the companies of the industrial sector. Today, the net salary of semi‐skilled workers is between US$ 200 and US$ 400 per month; supervisors earn between US$ 500 and US$ 800, and salaries of lower‐level managers range between US$ 1,000 and US$ 2,500. However, as we have seen, there are still important wage disparities between Western and Asian companies.

Driven for the most part by large industrial companies, the demand for Congolese skilled workers increased sharply between 2006 and 2008, and again between 2009 and 2011. Since then, it has remained low. In a context of declining copper prices, most companies have cut their workforce and limited the hiring of new workers to a minimum (on average, between 5 and 10 per year). With the exception of SCM, no new mining project has started production. In the past two years, copper and cobalt production has risen again, and activities in the mining sector have slowly resumed. It remains to be seen how this growth will translate into the labour market. The ups and downs of the past decade have made investors more cautious than in the 2000s.

### Local Unskilled Workers

Many permanent unskilled workers and day labourers are recruited on the local market. For unskilled workers, employers often use their staffs’ personal networks, while day labourers are usually taken on at the entrances to the plants or the mining concessions. In both cases, the selection of workers is generally left to the recruiting agent's discretion, but some mining companies that are established in rural areas have made the commitment to give preference to the ‘local community’ within the framework of their corporate social responsibility (CSR) programme. The companies’ social departments ask customary chiefs to propose a number of candidates. In the case of Tenke Fungurume Mining (TFM), which will be studied in more detail below, a database listing the name, skills and contact details of local residents was created before the start of the project. Until recently, low‐skilled workers were selected from this database by public draw.

Permanent unskilled workers and day labourers receive a salary of between US$ 100 and US$ 200 per month. In addition to this salary, permanent unskilled workers are legally entitled to various employment benefits, including transport to the mining site, holidays and access to healthcare for them and their family. Whereas in Chinese companies, legal benefits are not systematically awarded to workers, in Western companies, they are frequently supplemented by sacks of maize flour, school allowances and/or a year‐end bonus. Nevertheless, unskilled workers consider their remuneration to be insufficient to support their families. Most of them have to rely on credit and informal activities (such as driving a taxi or farming) in parallel with their job in order to make ends meet.

Unskilled workers also have more stable jobs than day labourers, who can be fired overnight without justification. Under Congolese labour law, day labourers cannot be hired for more than 22 days over a two‐month period. In artisanal mining companies, however, this limit is often exceeded. The former HR manager of an Indian mining project told me that the company employed between 250 and 800 labourers — mainly artisanal miners and handlers — on site for five years, before subcontracting the employment of this temporary workforce to a subsidiary company.^12^ Housed in makeshift tents, these labourers earned about US$ 5 per day and had to buy their own food. Every Sunday, a truck took them to the nearest market for this purpose.

Generally speaking, there are significant variations in the employment of day labourers and unskilled workers between artisanal and industrial mining companies, and between Asian and Western companies. As in the Indian project mentioned above, enterprises involved in artisanal mining hire a large number of day labourers as miners and/or handlers. In industrial mining projects, the number of day labourers is important in the construction phase, but falls sharply once production starts. With respect to permanent jobs, in contrast to Western companies, Asian companies involved in both artisanal and industrial mining tend to give preference to unskilled or low‐skilled workers, and to put them under the control of expatriate supervisors. This results in workers who are actually qualified for the role of supervisor, keeping their qualifications secret in the hope of being recruited. As explained above, however, tasks (such as catering, cleaning, bush clearing, etc.) that are performed by unskilled or low‐skilled workers have increasingly been outsourced to subcontractors in all of the mining companies. Today, whatever their size or national origins, mining companies directly employ only a limited number of unskilled workers and day labourers.

Overall, this analysis of mining companies’ hiring practices shows that such methods result in an ‘ethnotechnical hierarchy’ (Hecht, 2002: 699; see also Welker, 2014: 81), that correlates benefits of the job (such as stability, remuneration, working conditions, hierarchical position) with several of the workers’ social characteristics, particularly their language skills, level of education, place of residence and participation in various social networks. These hiring practices demonstrate different landscapes of labour for unskilled workers, national skilled workers and expatriates, and show how these three segments are respectively embedded in local communities, major Congolese cities and the countries where foreign investors are established. From a theoretical perspective, this three‐tiered geography of the labour market cannot be simply understood as resulting from the uneven distribution of linguistic, educational and social capital in space; it is also actively created by the people involved in the recruitment process and the social networks that they activate (Hanson and Pratt, 1992). As a result, the labour market has been rapidly caught in the factional struggles that make up Congolese political life at every level — what is commonly referred to as ‘geopolitics’ in the DRC.

## CONFLICTS

A common characteristic of the different segments of the mining sector labour market is the marginalization of women. Although Western companies give women a central place in their communication strategy, they only employ about 4 per cent of women, principally in non‐core departments (such as medical, social and HR). This percentage is likely to be even lower in Chinese companies, artisanal smelters and plants, or subcontracting companies, except in catering and cleaning. In artisanal mining, while there are many women sorting and washing heterogenite ore, they are excluded from mining sites, strictly speaking. So, in general, women are under‐represented in the mining sector, and the few who are employed tend to occupy secondary positions. As Francesca Pugliese's doctoral research^13^ shows, however, this marginalization does not seem to be a source of conflict in the Congolese copperbelt, where the mining industry has historically been constituted as a male preserve.

The same cannot be said for the inequalities between the different segments of the labour market identified above. These inequalities arouse strong feelings of injustice and dispossession, and give rise to more or less open conflicts. This will be illustrated below using the case of TFM, the most important mining project so far. Often cited as a model project due to its employment and community development policies, it has created particularly strong expectations in the Congolese population. During my stay in the TFM mining project in 2012, there were only 62 expatriates in a total workforce of 2,900. The use of words like ‘White’ or ‘Black’ was strictly forbidden, and some American workers made efforts to socialize with their Congolese colleagues and subordinates. In contrast to other Western companies, few complaints were filed with the HR department for racially discriminatory discourse or behaviour. However, it did not take long for Congolese managers to notice that many of the key positions were in the hands of expatriate (mostly American) executives, who were housed in a separate camp. The few Congolese in a position of responsibility were supervised by an expatriate, who monopolized relations with superiors (on the consequences of this practice on work relations, see Burawoy, 1972, 2014). One Congolese manager was particularly critical about the tendency of the Americans to ‘behave like chiefs’ — that is, to make decisions in a discretionary and authoritarian manner, without consulting Congolese colleagues — and to consider English language proficiency ‘a sign of intelligence’. For him, the way expatriate executives assess Congolese colleagues is only partially based on their work. As he explained, he gets up early and works hard, but he will never have access to the expatriates’ positions and remuneration.^14^

The privileges of expatriates may have resulted in individual frictions at work, but not in collective claims and conflicts as was the case with access to permanent jobs. Since the contract for the mining project was signed in 2005, the populations of neighbouring towns Tenke and Fungurume have tripled. Most immigrants, I was told, came with the hope of finding a job, even though a large number of them ended up working as artisanal miners. To stem the influx of these migrants and develop good relations with the ‘local community’, the company created a database listing the residents in the concession at the beginning of the project and set up a recruiting procedure giving priority to them. For low‐skilled jobs, candidates were to be selected from the database by public draw. Skilled jobs were open to all, but in the case of equal test results, candidates included in the database were to be given preference. On several occasions, the company also asked customary chiefs to send lists of candidates. Early on, however, voices were raised denouncing the recruitment of ‘foreigners’, demanding a say in the selection process, and calling for the establishment of quotas in favour of ‘residents’, ‘autochthons’, or the ‘children of chiefs’.

These terms clearly reflect the different meanings that have been attributed to the concept of ‘local community’ that is promoted by TFM. For chiefs, it refers to the community of their subjects, that is, Sanga people living inside the concession. For the cultural association Lwanzo Lwa Mikuba — which represents the interests of Sanga speakers — it refers to all the members of Sanga‐speaking tribes (Sanga, Yeke, etc.), whatever their place of residence. For Forces Vives, a platform representing the interests of various cultural associations in Katanga, it refers to people from Katanga Province residing in the concession, regardless of their tribe. These different meanings echo the flexibility of discourses about autochthony in DRC and elsewhere (Geschiere, 2009; Geschiere and Nyamnjoh, 2000; Hilgers, 2011; Jackson, 2006).

The company's social department reports on the situation in the local community and frequently notes the widespread discontent about the marginalization of ‘local’ people on the labour market. Among these dissenting voices, that of Lwanzo Lwa Mikuba has been the loudest. Its representatives have regularly sent letters, requested meetings and organized marches to denounce the grip of the Lunda people on the recruitment process, to demand the departure of some Lunda managers, and to recommend Sanga‐speaking workers.^15^ In a letter from 2012, written under the title ‘Operation Half‐and‐Half’, they even put forward a detailed plan that aimed to raise the proportion of ‘autochthons’ to 50 per cent of the company's workforce: it consisted of expelling foreigners from the company and replacing them with Sanga‐speaking people. Until now, TFM has challenged Lwanzo's figures, and has refused to meet its demands. In addition, with the development of the project, it has recruited fewer unskilled workers from the database, which has become increasingly obsolete. As a result, the ‘dialogue’ between the company and the cultural association has gradually deteriorated.

Finally, TFM witnessed an influx of artisanal miners to its concession. According to existing estimates, artisanal miners number between 3,000 and 10,000 in the area. They enter the concession during the night, with the complicity of the industrial guards and members of the mining police, and dig pits and tunnels in the hills. Each month hundreds of tons of heterogenite leave the concession in this way. However, security services sometimes arrest miners, seize their production, and hand them over for justice, which has caused violent confrontations on several occasions. In 2010, for example, a police team was dispatched to clear out artisanal miners from a hill: some miners were arrested and several trucks loaded with ore were seized. The following day, hundreds of miners walked to the TFM offices to claim access to the hill, a demand which was refused. In response, they looted and burnt several company trucks. In 2014, a similar incident occurred: an artisanal miner was killed during an altercation with the police and his fellow miners vandalized one of TFM's community liaison offices in retaliation.

As these news stories suggest, TFM's relationship with artisanal miners is more violent than that with Lwanzo. The main reason for this difference in treatment is that miners claim access to the ore, not to employment, and their opponents are not Congolese managers within the company, but the company itself. TFM regards artisanal miners as criminals who enter its property illegally, and so treats them as such. In turn, artisanal miners, who have nothing to lose, claim their rights to the land of their ancestors, and argue that the local economy benefits more from their activities than from the presence of TFM.

## CONCLUSION

The mining strategy of the World Bank has given rise to conflicting assessments of the potential contribution of foreign mining investments in Africa. Although important, this debate often leads to a depiction of African societies as being either the beneficiaries or the victims of new mining projects. At best, the consequences of the World Bank strategy are understood to result in a simple division between winners and losers. Focusing on the labour market in the Congolese copper mining sector, this article suggests that the mining boom has generated a more complex structure of inequalities.

Following the liberalization of the mining sector in the late 1990s and early 2000s, a large labour market was created as a result of the development of both industrial and artisanal mining, and the addition of dozens of new companies. This new market came to be segmented along three intersecting lines of division: the first corresponds to the distinction between industrial and artisanal mining companies; the second to the cleavage between direct jobs in mining companies and indirect jobs in subcontracting firms; and the third to the use of different channels for recruiting expatriate executives and supervisors, national managers and skilled workers, and local low‐skilled or unskilled workers. All these lines of structuration affect the quality of employment: each creates significant variations in terms of job stability, levels of compensation and working conditions. As a result, the labour market cannot be studied along one of these lines of division alone. Indirect jobs in subcontracting companies, for example, are not necessarily worse than direct jobs in mining companies. It depends on the type of company and the category of workers involved.

Particular attention has been paid to the third line of segmentation, and its embeddedness in space. The division of the labour market into three segments (for expatriates, managers and skilled workers, and unskilled workers) is often justified by the varying skills of these different categories of workers and the employment costs that are associated with them. From this viewpoint, their anchorage in different spaces (international, national and local) would simply reflect the uneven distribution of skills (and labour costs) in rural areas, large cities, and the expatriates’ countries of origin. A more detailed analysis, however, shows that these inequalities are also due to the mining companies’ use of separate recruitment channels, which mobilize different social networks. The search for a job is above all based on social networks, and for the three categories of workers involved, these networks are embedded in relatively separate places.

In view of the inequalities between the different segments of the labour market, it is hardly surprising that they cause resentment, friction and protest actions. To defuse potential conflicts, and secure their investment, some mining companies that take part in CSR initiatives have chosen to give priority to the ‘local community’ in the recruitment of personnel. As the case of TFM shows, this job localization strategy has not altered the three‐tiered segmentation of the labour market. However, it has given rise to claims from various collective actors, who attempt to control access to the labour market on the basis of autochthony, without much success. Their claims highlight the flexible nature of autochthony as a discourse: according to the speaker and the situation, it may refer to the chieftaincy, the locality, the tribe, the people speaking a given language, the province or the country. Whatever the size of the community that it calls upon, this discourse rests on a common feeling of dispossession, that of seeing the benefits of mining going to foreign entities, while the Congolese are left with the ‘holes’ — the abandoned quarries that the investors will leave behind at the end of their projects.

The labour market segmentation model that is sketched here could serve as a basis from which to compare the dynamics of inequality that are engendered by mining investments in other areas of the world, provided that particular attention is paid to the varying weight of the three lines of labour market segmentation mentioned above. The concomitant development of industrial and artisanal mining is contingent on geology. The importance of subcontracting depends on the degree to which production is mechanized, and on the presence of service companies locally. As for the segmentation of the labour market into separate recruitment channels, this varies with the location of mining projects, but the works of Hecht (2002) and Welker (2014) suggest that the ethnotechnical hierarchy found in DRC is not specific to this country, or to copper mining in general. In all circumstances, these cleavages are likely to cause tensions, and to give rise to claims for another form of wealth distribution on the basis of rights that are related to the land. Such claims are part of a struggle to change the rules of the game, but contrary to what Ferguson (2015) and Li (2017) seem to suggest, they do not necessarily contribute to more social justice. When expressed in the discourse of autochthony, ethnicity or nationalism — as is often the case, both in DRC and elsewhere — they are likely to result in other forms of exclusion and inequality.

## Footnotes

1 This industry is located in what is known as the Congolese copperbelt region, which corresponds to the new provinces of Haut‐Katanga and Lualaba (the southern part of the former Katanga province) along the border with Zambia.

2 With the exception of sociologists affiliated to the Society, Work, and Development Institute (SWOP) at the University of the Witwatersrand in South Africa (Bezuidenhout and Buhlungu, 2011; Forrest, 2015; Kenny and Webster, 1998), few researchers have taken the labour market as an entry point from which to study recent changes in the mining sector in Africa.

3 See: https://eiti.org/document/2014-democratic-republic-of-congo-eiti-report and https://eiti.org/document/republic-of-congo-2015-eiti-report. As the data in these EITI reports are based on the self‐reporting of mining companies, these figures must be taken with some caution. In addition, data are missing for several companies.

4 According to Jansson (2012: 182**–**83), the influx of these entrepreneurs was of the ‘gold‐rush’ type, which means that they were attracted by profit opportunities in the mining sector. For similar observations in Ghana and Cameroon, see Crawford and Botchwey (2017); Hilson et al. (2014); Nguepjouo (2017); Weng et al. (2015).

5 Interview, General Director of the Mining Cadastre, Kinshasa, 9 June 2017.

6 The SCM project is the result of a resource‐for‐infrastructure deal in 2009 between the Congolese government and a consortium of Chinese central state owned companies (see Jansson, 2013; Landry, 2018).

7 Based on interviews, CHEMAF manager, Lubumbashi, 9 and 13 November 2016, and 29 July 2017.

8 See https://eiti.org/document/republic-of-congo-2015-eiti-report

9 Since figures about indirect jobs are missing for many companies, their total number is certainly underestimated.

10 Interview, HR manager, Lubumbashi, 14 April 2017.

11 In most companies, the highest position held by a Congolese is that of HR manager, a position which is reserved for nationals by law.

12 Interview, former HR manager, Fungurume, 11 April 2017.

13 Pers. comm. Francesca Pugliese is working on her PhD thesis on mine workers in the Congolese copperbelt within the framework of the WORKinMINING project. Her main focus is on the experience of women working for foreign mining companies.

14 Interviews, TFM manager, Fungurume and Lubumbashi, 9 and 11 July 2012, and 6 August 2016.

15 Interviews with various Lwanzo representatives, Lubumbashi, Fungurume and Kolwezi, 1 August 2016, 12 April 2017 and 31 October 2017. Their demands must be understood in the broader context of the fragmentation and decentralization of the Congolese provinces that took effect in 2015 (see Englebert et al., 2018; Gobbers, 2016). Within the framework of this reform, the district of Kolwezi, where the most important mining projects are located, has been integrated into the province of Lualaba. According to Lwanzo representatives, this decision benefited the Lunda and other related tribes at the expense of the Sanga: Lunda leaders found themselves in a position to monopolize positions of power in the new provincial government, and to lay hands on the mining rent. From 2009 onwards, Lwanzo organized several protest actions against this decision, for which its national president was jailed for two years.
